# Phase III Trial of Albumin in Malaria Still Lacks Scientific Justification

**DOI:** 10.1371/journal.pctr.0020001

**Published:** 2007-02-09

**Authors:** Charles J Woodrow, Timothy Planche

We wrote to *PLoS Clinical Trials* [1] following the publication of the article by Akech et al. [2] in order to highlight specific problems in the design and analysis of the study presented, point out errors in the presentation of the data, and seek clarification over certain details. As a consequence of this letter an erratum [3] to the editorial commentary has been issued confirming that no difference was found in death rate or any other outcome measure between the two arms of this study.

The authors' follow-up letter [4] reiterates claims concerning the benefit of albumin over other fluids, including Gelofusine. Unfortunately, this letter missed an opportunity to clarify a number of issues and perpetuates a number of inaccuracies. For example, the notion of albumin's superiority over Gelofusine (groundless given the lack of statistical evidence for this; see erratum) persists in the statement beginning: “The combined findings that death, severe allergic reaction, and acute neurological events were more common in the Gelofusine group…” Similarly, the erroneous total patient number included in the meta-analysis (238) is still used in preference to the correct number (239).

Phase III studies must be based on specific and relevant phase II studies. These preferably assess intervention versus standard treatment (“maintenance-only” fluids in most African hospitals) and optimise dosing strategy while rigorously and proactively noting adverse events (e.g., pulmonary oedema by chest radiography [5]). However, none of the studies on albumin performed in Kilifi have incorporated these elements into their design and reported adverse events in a standardised fashion [5]. Even the amounts of fluid actually received by patients in the most recent study are not provided [2]. Comparison of case fatality rates with historical controls cannot provide the required safety data to underpin a phase III study. Additionally, given the lack of a clear hypothesis (the authors discuss albumin acting in both volume expanding and neuroprotective capacities), the group of patients who might benefit from albumin remains uncertain.

Failure to address any of the specific points in our letter impairs the ability of readers to review primary data for themselves. Repeating arguments for phase III studies on albumin in severe malaria does not make them more compelling.
